# Fibroblast growth factor 21, assisted by elevated glucose, activates paraventricular nucleus NUCB2/Nesfatin-1 neurons to produce satiety under fed states

**DOI:** 10.1038/srep45819

**Published:** 2017-04-04

**Authors:** Putra Santoso, Masanori Nakata, Kazuhiro Shiizaki, Zhang Boyang, Kumari Parmila, Zesemdorj Otgon-Uul, Koshi Hashimoto, Tetsurou Satoh, Masatomo Mori, Makoto Kuro-o, Toshihiko Yada

**Affiliations:** 1 Department of Physiology, Division of Integrative Physiology, Jichi Medical University School of Medicine, Yakushiji 3311-1, Shimotsuke, Tochigi 329-0498, Japan; 2 Division of Anti-aging Medicine, Center for Molecular Medicine, Jichi Medical University, Yakushiji 3311-1, Shimotsuke, Tochigi 329-0498, Japan; 3 Department of Molecular Endocrinology and Metabolism, Graduate School of Medical and Dental Sciences, Tokyo Medical and Dental University, Tokyo 113-8510, Japan; 4 Department of Preemptive Medicine and Metabolism, Graduate School of Medical and Dental Sciences, Tokyo Medical and Dental University, Tokyo 113-8510, Japan; 5 Department of Medicine and Molecular Science, Gunma University Graduate School of Medicine, 3-39-22 Showa-machi, Maebashi, Gunma 371-8511, Japan; 6 Metabolic and Obese Research Institute, Maebashi, Gunma 371-0037, Japan

## Abstract

Fibroblast growth factor 21 (FGF21), liver-derived hormone, exerts diverse metabolic effects, being considered for clinical application to treat obesity and diabetes. However, its anorexigenic effect is debatable and whether it involves the central mechanism remains unclarified. Moreover, the neuron mediating FGF21’s anorexigenic effect and the systemic energy state supporting it are unclear. We explored the target neuron and fed/fasted state dependence of FGF21’s anorexigenic action. Intracerebroventricular (ICV) injection of FGF21 markedly suppressed food intake in fed mice with elevated blood glucose. FGF21 induced c-Fos expression preferentially in hypothalamic paraventricular nucleus (PVN), and increased mRNA expression selectively for nucleobindin 2/nesfatin-1 (NUCB2/Nesf-1). FGF21 at elevated glucose increased [Ca^2+^]_i_ in PVN NUCB2/Nesf-1 neurons. FGF21 failed to suppress food intake in PVN-preferential Sim1-Nucb2-KO mice. These findings reveal that FGF21, assisted by elevated glucose, activates PVN NUCB2/Nesf-1 neurons to suppress feeding under fed states, serving as the glycemia-monitoring messenger of liver-hypothalamic network for integrative regulation of energy and glucose metabolism.

Fibroblast growth factor 21 (FGF21) is a circulating hormone produced predominantly in the liver^1^, and substantially in the adipose tissue^2^,^3^, skeletal muscle^4^, and pancreas^5^. Its amino acid sequence is highly identical between human and mouse^6^. It is suggested that FGF21 is released from the liver in response to fasting^1^ and high-fat, low-carbohydrate ketogenic diet in mice^7^ and to high-fat diet in monkeys^8^. In rodents, FGF21 mRNA expression in the liver is increased by 24 h fasting^1^,^9^, and circulating FGF21 level is also increased by 24 h fasting and reversed by refeeding^1^. Studies in humans also showed that circulating FGF21 is significantly increased by fasting for 10 days in healthy individuals^10^ or 7 days in patients with rheumatoid arthritis^11^. During fasting in mice, FGF21 is directly induced by peroxisome proliferator-activated receptor α (PPARα) in the liver, and in turn, FGF21 induces ketogenesis in liver and lipolysis in white adipose tissue (WAT)^12^. FGF21 also induces expression of peroxisome proliferator-activated receptor γ coactivator protein-1α (PGC-1α) in the liver and thereby promotes fatty acid oxidation, tricarboxylic acid cycle flux, and gluconeogenesis in fasted state^13^. These findings suggest that the hunger-associated hormone FGF21 serves to maintain the energy homeostasis by supplying energy substrates particularly under fasted conditions.

Against the postulated role of FGF21 to supply energy substrates, an FGF21 analog was recently shown to suppress food intake in obese monkeys followed by reduction of body weight^14^,^15^. However, in previous studies in obese rodents, FGF21 had no effect on feeding^16^ or apparently increased feeding^17^,^18^, showing inconsistency. Hence, the effect of FGF21 on feeding is debatable and whether it involves the central mechanism is less defined. Moreover, the effect of FGF21 on feeding in non-obese/diabetic subjects remains unclarified. To solve the apparent inconsistency in literature and thereby to establish the physiological relevance of FGF21, the present study explored the neuronal pathway mediating effect FGF21 on feeding and its dependency on metabolic states. We compared the effect of FGF21 on food intake under fasted and refed conditions, while monitoring corresponding blood glucose levels.

FGF21 is able to cross the blood-brain barrier by simple diffusion in mice^19^, and appears in the cerebrospinal fluids in humans^20^. Moreover, FGF21 receptors (fgfr1c, fgfr2c and fgfr3c) and their obligate coreceptor β-klotho are expressed ubiquitously in the central nervous system (CNS) including the hypothalamus, midbrain and hindbrain^21^. During starvation, FGF21 acts directly on the CNS, particularly the hypothalamus and hindbrain, to lower insulin and increase corticosterone levels in circulation and alter circadian behavior in mice^21^. FGF21 also remarkably reduces the preferences for sweet and alcohol in obese mice and the preference for sweet in obese monkeys, and these effects require β-klotho in the CNS in mice^22^. FG21 has been shown to increase phosphorylated extracellular signal-regulated kinase (p-ERK) expression in the paraventricular nucleus (PVN) of hypothalamus both *in vivo* and *in vitro* in mice^23^. Moreover, FGF21 is suggested as the hepatic signal that acts on the PVN to suppress sugar intake in mice^24^. These findings suggest that, besides its prominent activities in the peripheral tissues^25^,^26^,^27^,^28^, FGF21 also potentially acts on the CNS to elicit its effects^29^, and hypothalamus is one of the targets.

PVN is a feeding regulation center in the brain^30^. It consists of neurons expressing neuropeptides including arginine vasopressin (AVP), corticotropin-releasing hormone (CRH), oxytocin (Oxt), and nucleobindin-2/nesfatin-1 (NUCB2/Nesf-1), all of which have been implicated in feeding suppression^31^,^32^,^33^,^34^,^35^. It has been shown that FGF21 increases CRH mRNA expression in the brain including in PVN during fasting in mice^9^. However, whether FGF21 is also implicated in activation of other PVN neurons is less to be defined. Moreover, the functional relevance of the interaction between FGF21 and PVN neurons particularly in feeding regulation remains unknown. Cumulative evidence has indicated that NUCB2/Nesf-1 neurons in PVN play a role in suppressing feeding^34^,^35^,^36^,^37^,^38^, reducing body weight^35^,^39^, and increasing energy expenditure in rodents^40^. We reported that the NUCB2/Nesf-1 located in PVN regulates both total amount and circadian rhythm of food intake in rodents^34^,^38^,^41^. These functions regulated by PVN NUCB2/Nesf-1 largely overlap with those regulated by FGF21. However, the link of FGF21 to PVN NUCB2/Nesf-1 neurons is unknown. The present study aimed to specify the neurons targeted by and mediating the anorexigenic action of FGF21, with particular attention to PVN NUCB2/Nesf-1 neurons. Our data show that FGF21, being assisted by elevated glucose, specifically activates PVN NUCB2/Nesf-1 neurons to suppress food intake selectively under fed states.

## Results and Discussion

**Figure 1:**
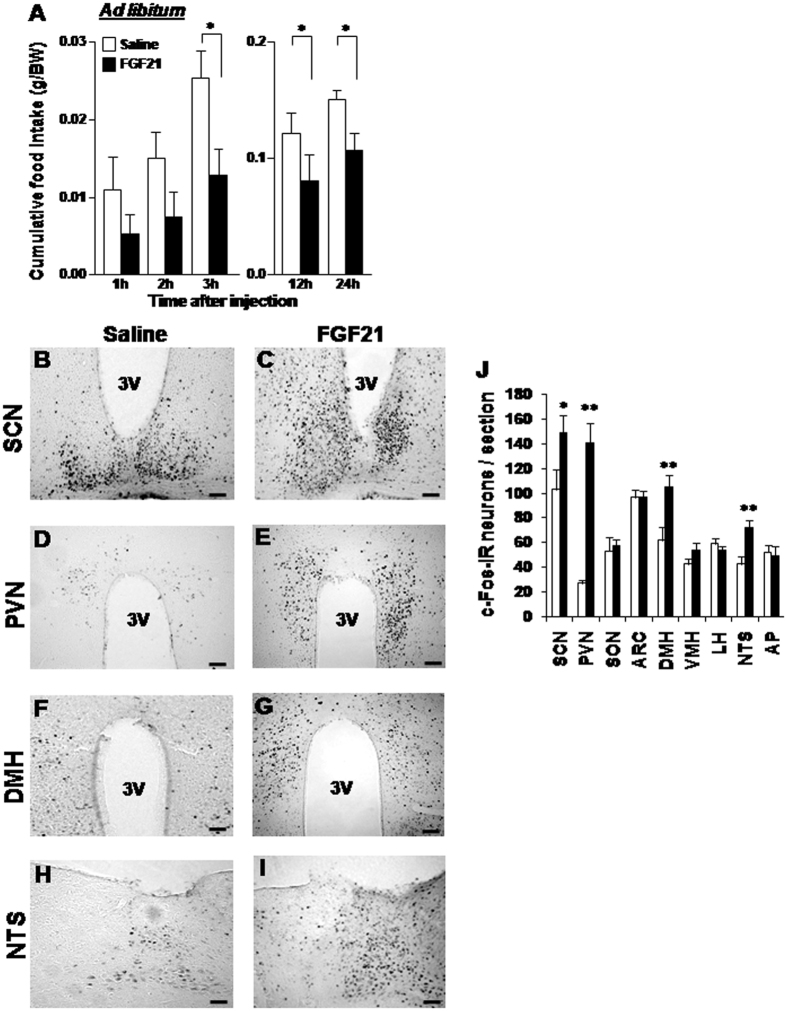
ICV injection of FGF21 reduces cumulative food intake and increases c-Fos expression in the hypothalamus and brain stem. (**A**) Cumulative food intake (g/BW) measured for 1 to 24 h after ICV injection of FGF21 (50 ng/2 μl) (black bars) or saline (white bars) in *ad libitum* groups. (**B**–**I**) Immunohistochemical staining of c-Fos in the SCN, PVN, DMH and NTS after injection of FGF21 (right panels) or saline (left panels). (**J**) Number of c-Fos-IR neurons per section in the hypothalamic and brain stem regions. Data are presented as mean ± SEM. *P < 0.05, **P < 0.01 by two-way ANOVA followed by Bonferroni post-hoc test in (**A**,**B**) and Student’s t-test in (**J**). n = 5–6 in (**A**) and 3 in (**J**). Scale bars represent 200 μm.

In this study, FGF21 was injected right before the onset of the dark phase in *ad libitum* fed mice, whose blood glucose level was 130.5 ± 6.8 mg/dl. The intracerebroventricular (ICV) injection of 50 ng FGF21 in 2 μl saline, compared to saline control, tended to decrease cumulative food intake at 1 and 2 h and significantly reduced it at 3, 12 and 24 h after injection (Fig. 1A). FGF21 at a lower dose of 10 ng did not alter food intake at 24 h, exhibiting a dose-dependent effect (Supplemental Fig. 1).

To explore the brain region targeted by FGF21, we investigated c-Fos expression in the areas of the hypothalamus and brain stem implicated in feeding. ICV injection of FGF21 significantly increased c-Fos expression in the suprachiasmatic nucleus (SCN), PVN, dorsomedial hypothalamus (DMH), and nucleus tractus solitarius (NTS) (Fig. 1B–J), confirming previous report that FGF21 increased c-Fos in PVN^24^. Among these four regions, the increment of c-Fos expression was outstandingly high (five-fold) in PVN (Fig. 1J), suggesting that the anorexigenic effect of FGF21 could involve PVN.

**Figure 2:**
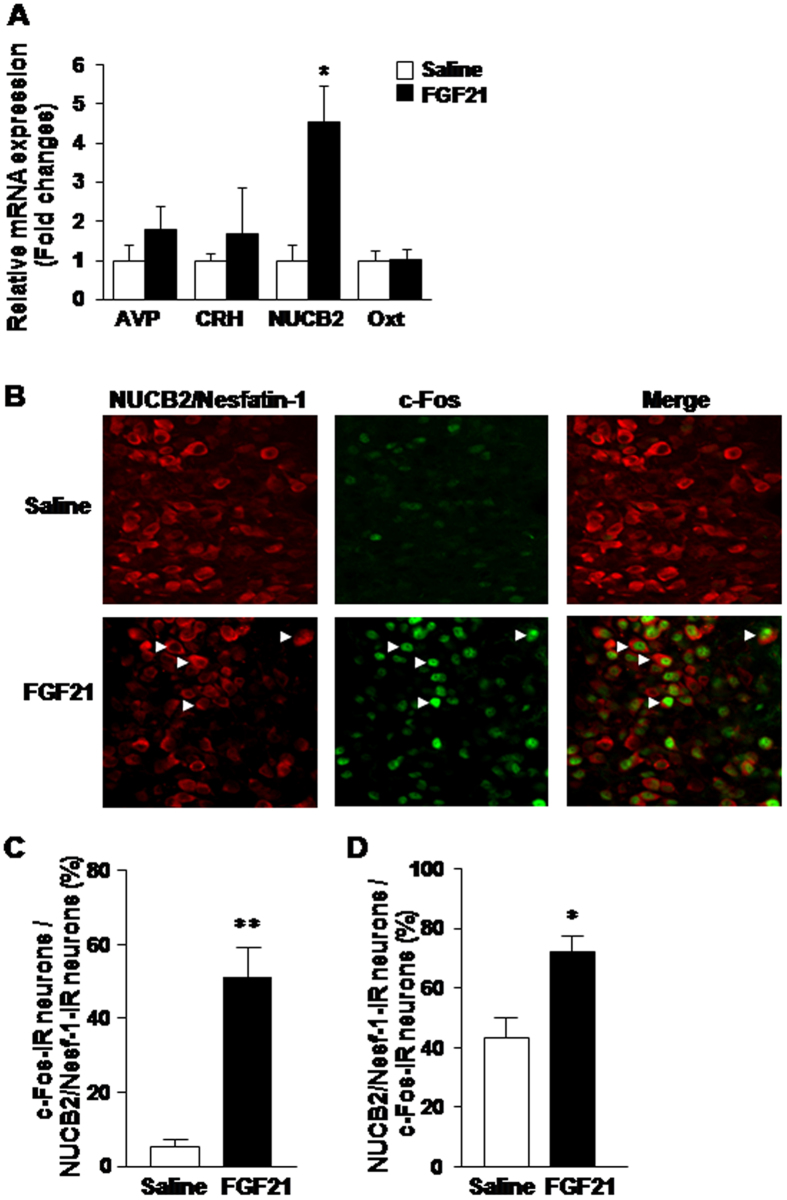
ICV injection of FGF21 increases NUCB2 mRNA expression and c-Fos expression in NUCB2/Nesf-1 neurons of PVN. (**A**) Relative mRNA expression (fold change) of AVP, CRH, NUCB2, and Oxt in PVN after ICV injection of saline (white bars) or FGF21 (50 ng/2 μl) (black bars) at 18:30. (**B**) Double immunofluorescence staining of c-Fos and NUCB2/Nesf-1 in PVN after ICV injection of saline or FGF21. (**C**,**D**) Percentage of c-Fos-IR neurons in NUCB2/Nesf-1-IR neurons (**C**), and percentage of NUCB2/Nesf-1-IR neurons in c-Fos-IR neurons (**D**) at 2 h after injection of saline (white bars) or FGF21 (black bars). White arrows in B indicate the neurons IR to both NUCB2/Nesf-1-and c-Fos. Data are presented as mean ± SEM. **P < 0.01, *P < 0.05 by Student’s t test. n = 4 animals per group.

To specify the PVN neuron subpopulation targeted by FGF21, the expression of neuropeptides and c-Fos in PVN after ICV injection of FGF21 was examined. ICV administration of FGF21 increased mRNA expression of NUCB2/Nesf-1, but not AVP, CRH and Oxt (Fig. 2A). In the experiments of double immunofluorescence staining for c-Fos and NUCB2/Nesf-1 in PVN (Fig. 2B), administration of FGF21 increased the incidence of c-Fos- immunoreactive (IR) neurons among NUCB2/Nesf-1-IR neurons 8.5-fold (Fig. 2C), and that of NUCB2/Nesf-1-IR neurons among c-Fos-IR neurons 1.7-fold (Fig. 2D). Thus, FGF21 markedly induced c-Fos expression in NUCB2/Nesf-1-IR neurons, indicative of activation of NUCB2/Nesf-1 neurons in PVN. In our previous study, the incidence of NUCB2/Nesf-1-IR neurons in PVN was increased by refeeding^42^. Hence, in the present study, we examined the expression of NUCB2/Nesf-1 in fasted condition to exclude the effect of food consumption and selectively observe the effect of FGF21. The results of the present study indicate that FGF21 preferentially targets NUCB2/Nesf-1 in PVN. Previous study, however, reported that FGF21 increases CRH mRNA expression in the hypothalamus^9^. This apparent discrepancy could be due to different doses of FGF21: the previous study used approximately eight times higher dose than our study. At the lower dose used in our study, FGF21 may preferentially activate NUCB2/Nesf-1 expression, but at the higher dose used in the previous study^9^ it could activate CRH expression. Alternatively, the increase of CRH expression could take place later than that of NUCB2/Nesf-1, and is missing in the time frame of measurements in our study.

**Figure 3:**
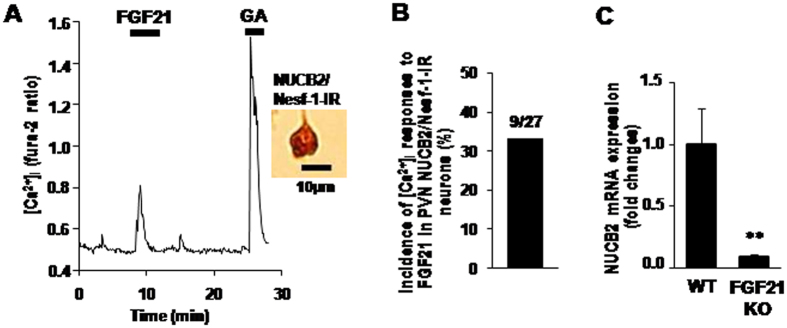
FGF21 increases [Ca^2+^]_i_ in single PVN NUCB2/Nesf-1 neurons and endogenous FGF21 regulates PVN NUCB2/Nesf-1 mRNA expression. (**A**) Addition of 50 ng/ml FGF21 for 5 min to superfusate increased [Ca^2+^]_i_ in a single PVN neuron, which was subsequently shown to be IR to NUCB2/Nesf-1 by immunocytochemical staining. [Ca^2+^]_i_ is expressed by fura-2 fluorescence ratio (F340/F380). Superfusate was KRB containing 5.6 mmol/l glucose. This neuron also responded to 10^−6^ M glutamic acid (GA) with an increase in [Ca^2+^]_i_. (**B**) Incidence of [Ca^2+^]_i_ responses to FGF21 in PVN NUCB2/Nesf-1 neurons, presented by percentage. The number above the bar indicates the number of neurons that responded over that examined. (**C**) Relative mRNA expression (fold change) of PVN NUCB2/Nesf-1 in wild-type and Fgf21 KO mice. Data are presented as mean ± SEM. **P < 0.01 by Student’s t-test. n = 4–6 in (**C**).

To investigate whether FGF21 activates PVN NUCB2/Nesf-1 neurons by a direct action, we measured cytosolic calcium concentration ([Ca^2+^]_i_) in the single neurons isolated from PVN. Administration of FGF21 (50 ng/ml) increased [Ca^2+^]_i_ in the PVN neurons that were subsequently shown to be IR to NUCB2/Nesf-1 (Fig. 3A). Nine of 27 PVN NUCB2/Nesf-1 neurons (33%) responded to FGF21 with [Ca^2+^]_i_ increases (Fig. 3B). These results suggested that FGF21 is a direct and prominent regulator of NUCB2/Nesf-1. Furthermore, in fgf21 knock out (KO) mice, NUCB2/Nesf-1 mRNA expression in PVN in light phase was markedly reduced compared to wild-type (WT) mice (Fig. 3C), suggesting that the endogenous FGF21 maintains NUCB2/Nesf-1 in PVN. These data collectively indicate that FGF21, as a physiological regulator, directly interacts with the PVN NUCB2/Nesf-1 neuron to raise its neuronal activity and mRNA expression. As the molecular basis for this interaction, the FGF21 receptors, fgfr1c, fgfr2c and fgfr3c, and its co-receptor β–klotho, are all expressed in PVN^21^.

**Figure 4:**
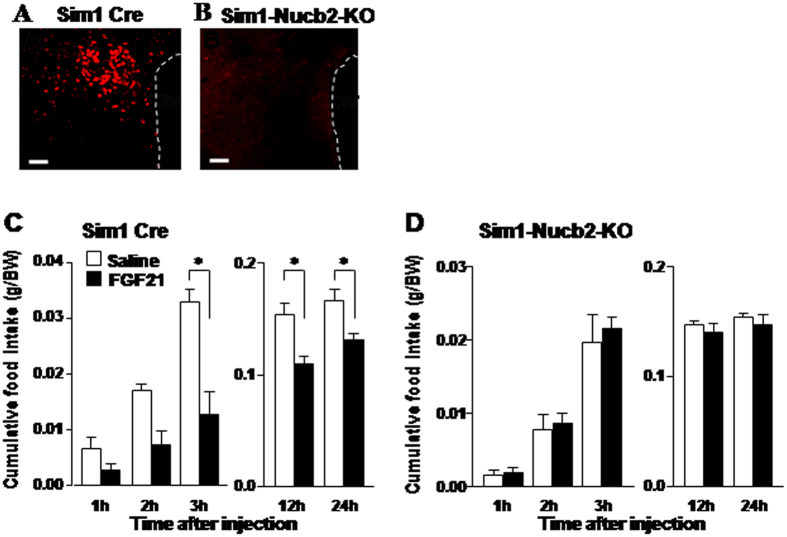
Knockout of NUCB2 in PVN abolishes the anorexigenic effect of FGF21. Immunofluorescence pictures of PVN NUCB2/Nesf-1 in Sim1 Cre mice (**A**) and Sim1 Cre-Nucb2-KO mice (**B**). Scale bars represent 100 μl. Cumulative food intake (g/BW) in Sim1 Cre mice (**C**) and Sim1-Nucb2-KO mice (**D**) for 1, 2, 3, 12 and 24 h after injection of saline (white bars) or 100 ng/2 μl FGF21 (black bars). Data are presented as mean ± SEM. *P < 0.05 by two-way ANOVA followed by Bonferroni post-hoc test. n = 4–7 in (**C**,**D**). See also Supplemental Figs 2 and 3.

To explore whether PVN NUCB2/Nesf-1 mediates the anorexigenic action of FGF21, we generated the PVN Nucb2 KO mice by crossing Single minded-1 (Sim1) Cre mice and Nucb2^flox/flox^ mice (Sim-1-Nucb2-KO mice) (Supplemental Fig. 2A,B). Sim1 gene is expressed predominantly in the PVN and supraoptic nucleus (SON)^43^. In the Sim-1-Nucb2-KO mice, expression of immunofluorescence for NUCB2/Nesf-1 was absent in PVN (Fig. 4A,B) and SON (Supplemental Fig. 3A,B), whereas it was observed in SCN, arcuate nucleus, and DMH with similar intensities as in control Sim-1 Cre mice (Supplemental Fig. 3C–H). These results confirmed the specific KO of NUCB2/Nesf-1 in PVN and SON. In control Cre mice, ICV injection of FGF21 (50 ng/2 μl), compared to saline, significantly reduced cumulative food intake at 3, 12, and 24 h after injection (Fig. 4C). In KO mice, in contrast, ICV injection of FGF21 failed to significantly alter food intake at all time points after injection (Fig. 4D). These results suggest the NUCB2/Nesf-1 in PVN and/or SON to be the major target for the anorexigenic effect of FGF21. FGF21 at this dose was found to induce c-Fos in PVN but not SON (Fig. 1J). These results, taken together, support that the PVN NUCB2/Nesf-1 mediates the anorexigenic action of FGF21.

**Figure 5:**
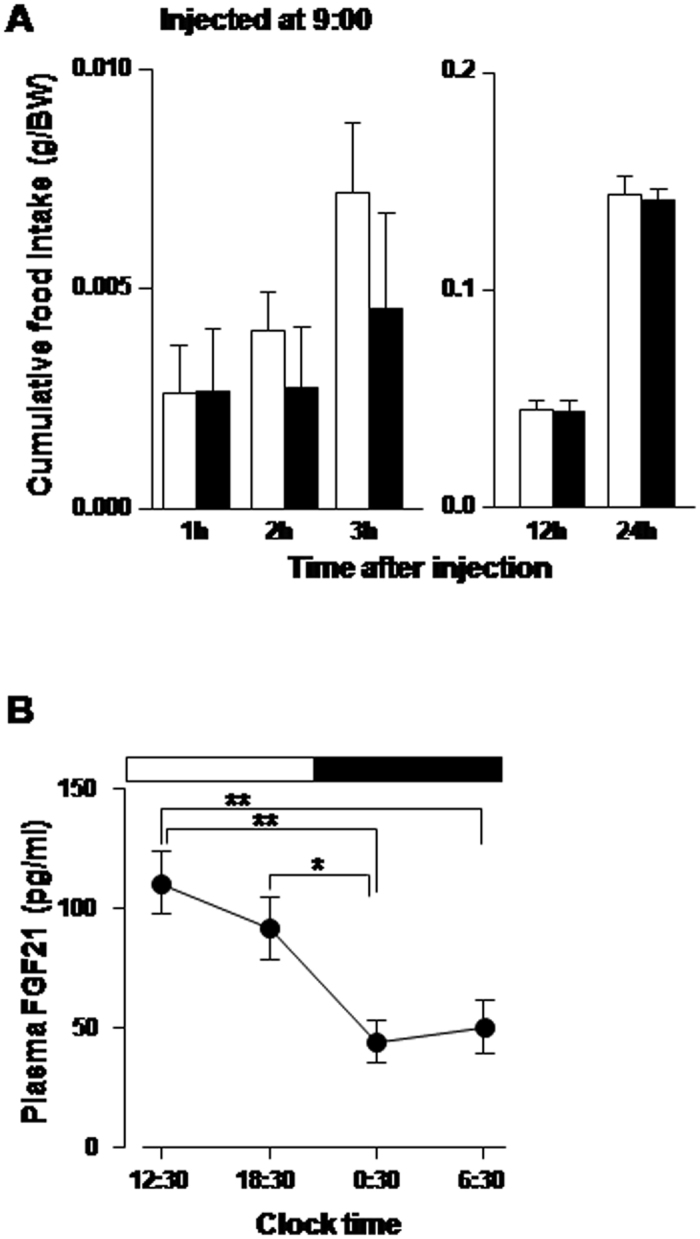
ICV injection of FGF21 at early light phase did not significantly reduce food intake in mice. (**A**) Cumulative food intake (g/BW) after ICV injection of FGF21 (50 pmol; black bars) or saline (white bars) at 09:00. (**B**) Plasma FGF21 concentration (pg/ml) in *ad libitum* mice showing the elevated levels during light phase and lowered levels during dark phase. **P < 0.01, *P < 0.05. n = 6 in A and 7 in (**B**).

Our current result demonstrating the anorexigenic effect of FGF21 injected at the onset of dark phase is apparently contradictory with the previous reports. Peripheral injection of FGF21 did not reduce food intake in lean mice and rats^24^,^44^, and overexpression of FGF21 also did not reduce food intake in lean mice^45^. Moreover, chronic intracerebroventricular (ICV) injection and peripheral administration of FGF21 increased or did not alter daily food intake in obese mice and rats^16^,^17^,^18^. To solve the apparent discrepancy between the present and previous studies, we examined the effect of ICV FGF21 injection at the early light phase (09:00) on feeding in normal mice. FGF21 injection at the early light phase failed to suppress food intake (Fig. 5A). Hence, it may suggest that the anorexigenic effect of FGF21 depends on the time of injection. We previously reported that expression of NUCB2/Nesf-1 in PVN, which mediates the FGF21 action, is elevated in light phase and lowered in the dark phase^41^. Accordingly, FGF21 may be less effective if injected in light phase when NUCB2/Nesf-1 level is already elevated, and more effective if injected in dark phase when NUCB2/Nesf-1 level is reduced. Moreover, plasma FGF21 level was significantly higher in light phase (12:30, 18:30) than in dark phase (0:30, 6:30), showing diurnal rhythm (Fig. 5B). Consequently, the injection of FGF21 in light phase may be less effective, since the elevated endogenous FGF21 already exerts the metabolic effect. Furthermore, it has been reported that the half-life of FGF21 activity is relatively short (maximum 2 h) in mice^46^, and that intravenous injection of FGF21 could easily reach the brain but is quickly degraded: the concentration drops off to approximately 41% within 20 min in brain tissue of mice^19^. Thus, it is expected that a large part of FGF21 injected in early light phase is degraded before it can exert anorexigenic effect later in dark phase when feeding is profoundly promoted. These reports and our present finding, taken together, suggest that the essential condition for FGF21 to be anorexigenic is the injection timing in dark phase when endogenous FGF21 activity is lowered. The apparent discrepancy could also be due to differences in the FGF21 dose and experimental animals as well as the time window for food intake measurements. We used a lower dose of FGF21 (50 ng) than previous study (400 ng)^17^, and measured food intake from 1 h through 24 h after injection while the previous studies measured only daily food intake^16^,^17^,^18^,^44^. Furthermore, we used normal mice, while the previous studies^16^,^17^,^18^ used obese rodents (DIO or ob/ob) that may exhibit impaired leptin system, insulin action and glycemia^47^, which may have resulted in different responsiveness to FGF21. A study in mice has suggested that obesity is associated with FGF21-resistant state^48^, which may also explain the discrepancy in feeding response to FGF21 treatment between obese and normal animals. Regarding the discrepancy between our result and the previous report that overexpression of FGF21 did not decrease feeding in lean mice^45^, it may be due to downregulation of anorexigenic hormone leptin. It has been reported that plasma leptin was significantly lower in transgenic mice overexpressing FGF21 as compared to normal mice^44^. Since leptin is implicated in feeding suppression, its low level may lead to the insufficient effect of overexpression of FGF21 on feeding. However, further study is definitely required to address this issue.

In the case of fgf21 KO, we found that there was no difference in daily food intake between KO and wild-type (WT) mice under *ad libitum* condition (data not shown). The result does not apparently fit with the finding that fgf21 KO mice exhibit impaired NUCB2/Nesf-1 expression in PVN. It has been reported that daily food intake (g/day) in fgf21 KO mice was significantly higher than that in WT mice under both *ad libitum* and calorie restriction regimes under normal diet condition^49^. However, another report showed that daily food intake (kcal/day) was comparable between fgf21 KO vs. WT mice under normal diet condition^50^. This discrepancy suggests that under some conditions in KO mice, other anorexigenic factors may compensate the lack of NUCB2/Nesf-1 in PVN and thereby maintain normal feeding behavior. However, this issue definitely requires further investigation.

**Figure 6:**
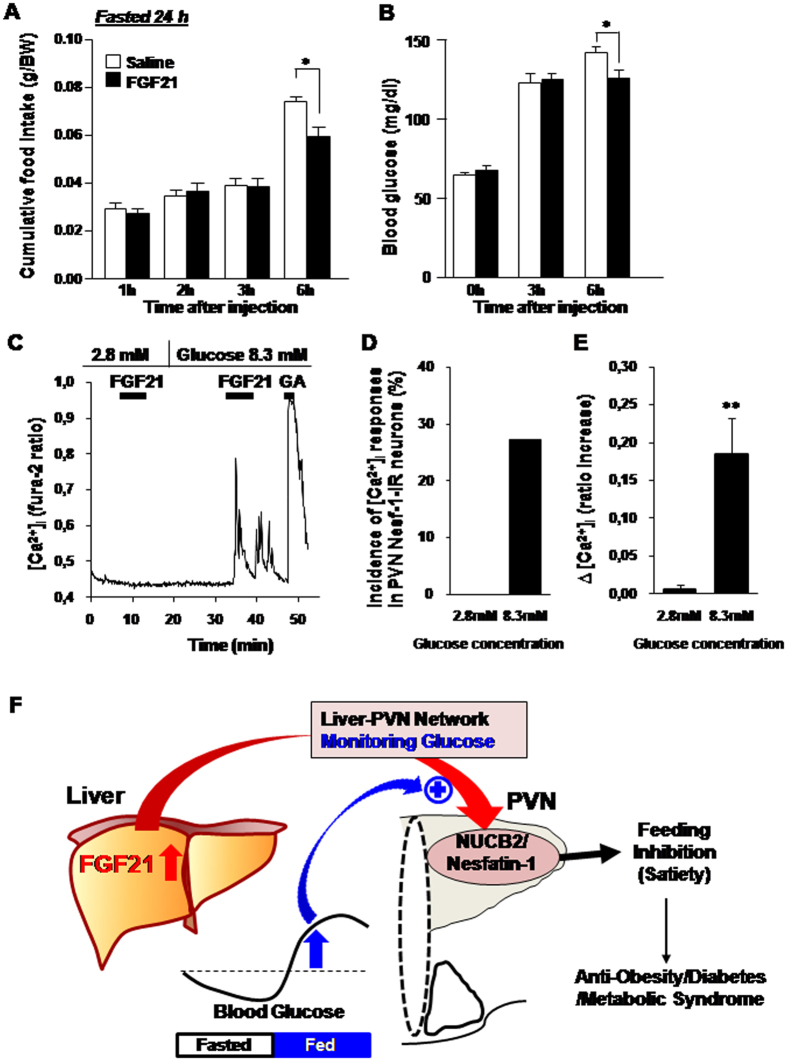
Fed state or elevated glucose, but not fasted state or lowered glucose, admits the effect of FGF21 on food intake and neuronal activity. (**A**,**B**) Food intake and blood glucose in mice ICV injected with FGF21 and refed after 24 h fasting. (**A**) ICV injection of FGF21 (black bars), compared to saline (white bars), did not significantly alter cumulative food intake (g/BW) up to 3 h, but suppressed it for 3~6 h period after injection and refeeding. (**B**) The blood glucose level was measured at 0, 3 and 6 h after injection and refeeding. It started to increase at 3 h after injection and refeeding. *C-E*, Effect of FGF21 at low and elevated glucose on [Ca^2+^]_i_ in PVN NUCB2/Nesf-1 neurons. (**C**) Administration of 50 ng/ml FGF21 with 2.8 mM (50 mg/dl) glucose failed to increase [Ca^2+^]_i_, while with 8.3 mM (150 mg/dl) glucose it markedly increased [Ca^2+^]_i_ in single NUCB2/Nesf-1 neurons isolated from PVN. FGF21 was added to superfusate for 5 min. This neuron also responded to 10^−6^ M glutamic acid (GA) with increases in [Ca^2+^]_i_. These data are representative of 6 neurons. (**D**) Incidence of [Ca^2+^]_i_ responses to FGF21 in PVN NUCB2/Nesf-1 neurons at 2.8 mM and 8.3 mM glucose, expressed by percentage. (**E**) Mean amplitude of [Ca^2+^]_i_ changes in response to FGF21 at 2.8 and 8.3 mM glucose, expressed by ratio (F340/F380) increase. (**F**) Schematic model for the FGF21-NUCB2/Nesf-1 axis that serves as the messenger for the liver-hypothalamic network and systemic glucose sensor for integrative regulation of feeding and metabolism. Data are presented as mean ± SEM. *P < 0.05, **P < 0.01 by two-way ANOVA followed by Bonferroni post hoc test in (**A**,**B**) and Student’s t-test in (**E**). n = 3–6 in (**A**,**B**).

The previous report that FGF21 is released in response to fasting and the present result that FGF21 suppresses food intake, are inconsistent for appropriate energy supply in fasted condition. A study in mice showed that the FGF21 present in the plasma functions during refeeding and that FGF21 is also produced by the liver in fully fed states and functions as an insulin sensitizer^1^. Here, in further, we explored whether the anorexigenic effect of FGF21 on food intake and neuronal activity depend on fasted vs. fed conditions or blood glucose levels. The results showed that in mice ICV injected with FGF21 and refed after 24 h fasting, FGF21 had no effect on cumulative food intake for up to 3 h, but significantly reduced food intake for 3~6 h time period after injection and refeeding (Fig. 6A). Moreover, blood glucose level (Fig. 6B) was as low as 66.7 ± 2.2 mg/dl at the time of injection and elevated to 125~126.5 mg/dl at 3 h after injection and refeeding in both FGF21 and saline groups. The elevation of blood glucose was significantly attenuated at 6 h in FGF21 injected group as compared to saline group. The elevation of blood glucose observed at 3 h after injection in FGF21 injected and control groups may originate from the glucose obtained from ingested food. The attenuation of blood glucose elevation at 6 h in FGF21 injected group might be due to feeding suppression during 3~6 h by FGF21. Taken together, these results indicate that single ICV injection of FGF21 does not suppress feeding in fasted states and initial refed period with blood glucose levels lower than the basal, but suppresses feeding in fed states with elevated blood glucose. In single cell analysis, concomitantly, FGF21 at 2.8 mM (50 mg/dl) glucose failed to increase [Ca^2+^]_i_, but at 8.3 mM (150 mg/dl) glucose it markedly increased [Ca^2+^]_i_ in the same PVN NUCB2/Nesf-1 neurons (Fig. 5C). Six of 22 PVN NUCB2/Nesf-1 neurons (27.3%) responded to FGF21 at 8.3 mM, but none of them (0%) at 2.8 mM (Fig. 6D and E).These results demonstrate that FGF21 exhibits anorexigenic effects selectively under fed conditions with elevated blood glucose concentrations (Fig. 6F). It was previously reported that plasma FGF21 is elevated in mice after refed with low protein-high-carbohydrate diet, and that FGF21 mRNA expression is markedly elevated under high glucose concentration in the presence of insulin in rat hepatocytes *in vitro*^51^. These finding may support that elevated glucose level is essential for induction of FGF21. These previous reports and our current results, taken together, suggest that elevated glucose associated with food ingestion promotes both FGF21’s induction and action on NUCB2/Nesf-1 neurons, thereby suppressing food intake. The present study revealed FGF21 to be the physiological upregulator of the PVN NUCB2/Nesf-1. It has been shown that the NUCB2/Nesf-1 expression in PVN exhibits circadian rhythm featured with a rise in light period (LP) in rodents^41^, and that it serves to produce circadian feeding rhythm and to restrict LP food intake^38^,^41^. Moreover, in both obese rodent models and PVN NUCB2 knockdown mice, the loss of circadian rhythm of PVN NUCB2/Nesf-1 expression leads to impaired circadian feeding rhythm and hyperphagia^38^,^41^, both of which trigger obesity and metabolic syndrome. Hence, the present results suggest a novel strategy to treat obesity: supplementation of FGF21 could restore endogenous PVN NUCB2/Nesf-1 expression and thereby reconstruct circadian feeding rhythm to ameliorate obesity and metabolic syndrome.

It has been shown in rodent models, FGF21 decreased glycemia and lipidemia, improved insulin sensitivity, increased energy expenditure and reduced body weight^16^,^17^,^18^,^52^. Another study showed that hyperglycemia in type 1 diabetic mice was counteracted by the glucagon receptor-deficiency via mechanisms involving marked compensatory upregulation of FGF21^53^. *In vitro* study revealed that FGF21 did not induce proliferation of various cell types^42^, suggesting the lack of mitogenic ability as a side effect. Studies in monkeys and humans have recently shown that an FGF21 analog, PF-05231023, reduced food intake and body weight in obese monkeys and obese/overweight humans with type 2 diabetes^14^. Hence, FGF21 has a potential therapeutic role to treat metabolic syndromes including diabetes and obesity^54^,^55^,^56^,^57^,^58^. In spite of these findings, the mechanisms underlying these anti-diabetic and anti-obese effects of FGF21 have remained less defined. Our study provides a novel mechanism that FGF21, being assisted by elevated glucose levels, specifically activates PVN NUCB2/Nesf-1 neurons to suppress food intake selectively under fed states with elevated blood glucose (Fig. 5F). Hypothalamic NUCB2/Nesf-1 was shown to regulate the glucose metabolism in the liver^59^. Therefore, the FGF21 to NUCB2/Nesf-1 axis might serve as the messenger for the liver-hypothalamic network and systemic glucose sensor for the integrative regulation of feeding, energy and glucose metabolism, possibly acting against obesity, diabetes and metabolic syndrome (Fig. 6F). This finding helps understand the central action of FGF21 and provides a strong mechanistic basis for the further clinical use of FGF21 and its analogs to treat obesity, diabetes, and metabolic syndrome.

## Materials and Methods

### Animals

Male C57BL/6 mice (SLC, Hamamatsu, Japan) were single-housed for *in vivo* experiments and group-housed for Ca^2+^ imaging under a 12-h light/dark cycle condition (7:30 light on). Food (Standard animal chow CE-2; CLEA, Osaka, Japan) and water were available *ad libitum* except particular experiments performed under fasted conditions. Sim1 Cre mice^60^ (a generous gift from Dr. Joel K. Elmquist, University of Texas), fgf21-KO mice^61^ (kindly provide from Dr. Steve Kliewer, University of Texas), Nucb2-floxed mice, and Sim1-Nucb2-KO mice were maintained in the same conditions as for C57BL/6 mice. The Sim1-Nucb2-KO mice were generated based on procedures described in Supplemental method and Supplemental Fig. 2A and B. All animal procedures were conducted in compliance with protocols approved by Jichi Medical University Animal Care and Use Committee, and all experiments were carried out in accordance with the approved protocols.

### Intracerebroventricular (ICV) injection

In mice aged 10 weeks, a guide cannula (ICM-23G09; Intermedical, Osaka, Japan) was inserted into lateral ventricle with the tip located at 0.5 mm caudal, 0.1 mm lateral to the bregma, and 2.2 mm below the skull. Mice were allowed to recover from surgery for at least one week before being subjected to tests.

For feeding experiments, mouse FGF21 (R & D System, Inc., Minneapolis, MN, USA) were dissolved in sterile saline and ICV injected at 10 ng/2 μl or 50 ng/2 μl. Animals were fasted for 2 h prior to injection and refed immediately upon injection. The injections were performed 1 h before the onset of dark cycle (18:30) and food intake was measured for 1, 2, 3, 12, and 24 h after injection. In other groups, animals were fasted for 24 h before the ICV injection of FGF21 (50 ng/2 μl; 09:00) followed by refeeding and food intake measurement from 1 to 24 h after injection.

For mRNA expression and c-Fos experiments, animals were also fasted 2 h followed by ICV injection of FGF21 at 18:30, but without refed after injection.

### Measurement of blood glucose

Blood was collected from *ad libitum* fed, 24 h fasted and 6 h refed mice, and blood glucose concentration was measured by glucocard (Arkay, Kyoto, Japan)^38^.

### Measurement of plasma FGF21

Blood was collected in tubes containing EDTA and aprotinin, and separated by centrifugation for 10 min at 3000 g. Platelet-poor plasma samples were collected and stored at −80 °C. Plasma FGF21 levels were measured using ELISA kits (R&D System, Inc., Minneapolis, MN, USA).

### RT-PCR

RT-PCR was performed as previously reported^62^. Briefly, at 2 h after ICV injection of FGF21, bilateral portions of PVN were punched out from hypothalamic slices. Quantitative RT-PCR assay was performed using SYBR Premix Ex Taq II polymerase in Thermal Cycler Dice (Takara bio). Expression levels of mRNAs were calculated by the ΔΔCT method of relative quantification, and normalized to products glyceraldehyde-3-phosphate dehydrogenase (GAPDH). The primers used in this experiment were listed in Supplemental Table 1.

### c-Fos expression

c-Fos expression was examined by the procedures described previously^34^,^42^. Briefly, at 2 h ICV injection of FGF21, animals were perfused transcardially using phosphate buffer containing 4% paraformaldehyde and 0.2% picric acid. The brain was sampled and proceeded for c-Fos immunostaining using rabbit anti-c-Fos anti-serum (sc-52; Biotechnology Inc., Santa Cruz, California, USA; 1:2000 dilution) and biotinylated goat-anti-rabbit IgG (Vector Laboratories Inc., Burlingame, California, USA; 1:500) as the primary and secondary antibodies, respectively. In double immunofluorescence staining of c-Fos and NUCB2/Nesf-1, mouse anti-c-Fos (ABCAM, Cambridge; 1:1000) and rabbit anti-NUCB2 (Sigma-Aldrich, St. Louis, Missouri; 1:1000) were used as the primary antibodies. Alexa fluor 488 goat anti-mouse (Life Technologies, Carlsbad, CA; 1:500) and Alexa fluor 594 donkey anti-rabbit (Life Technologies, Carlsbad, CA; 1:500) were used as the secondary antibodies for c-Fos and NUCB2, respectively. The confocal fluorescence images for c-Fos and NUCB2/Nesf-1 were acquired using Olympus FV1000 confocal laser-scanning microscope (Olympus, Tokyo, Japan)^34^.

### Immunofluorescence staining of NUCB2/Nesf-1

Brain samples were stained for NUCB2/Nesf-1 following previous report^42^ with slight modifications. Briefly, rabbit anti-NUCB2 (Sigma-Aldrich, St. Louis, Missouri; 1:1000) was used as the primary antibody and Alexa fluor 594 donkey anti-rabbit as the secondary antibody.

### Measurement of [Ca^2+^]_i_ and NUCB2/Nesf-1 immunocytochemistry in single PVN neurons

Single neurons were prepared from mice aged 5–6 weeks according to procedures reported previously^34^. [Ca^2+^]_i_ was measured by ratiometric fura-2 fluorescence imaging. Briefly, single neurons on coverslips were incubated with 2 μmol/l fura-2/AM (Dojin chemical, Kumamoto, Japan) for 40 min at room temperature, mounted in chamber, and superfused with HEPES-buffered Kreb-Ringer bicarbonate buffer (HKRB) composed of (in mM) 129 NaCl, 5.0 NaHCO_3_, 4.7 KCl, 1.2 KH_2_PO_4_, 1.8 CaCl_2_, 1.2 MgSO_4_, 10 HEPES and 5.6 glucose at pH 7.4. The fluorescence images due to excitation at 340 and 380 nm were captured and ratio (F340/F380) images produced by Aquacosmos system (Hamamatsu Photonics, Hamamatsu, Japan). The single neurons subjected to [Ca^2+^]_i_ measurements were subsequently immunostained for NUCB2/Nesf-1 using anti-NUCB2 antibody as previously described^34^.

### Statistical analysis

Data were presented as mean ± SEM. The two-tailed unpaired Student’s t-test was applied for two groups and two-way ANOVA for multiple groups. Post-hoc multiple comparison was generated using Bonferroni test. P < 0.05 was considered significant.

## Additional Information

**How to cite this article**: Santoso, P. *et al*. Fibroblast growth factor 21, assisted by elevated glucose, activates paraventricular nucleus NUCB2/Nesfatin-1 neurons to produce satiety under fed states. *Sci. Rep.* **7**, 45819; doi: 10.1038/srep45819 (2017).

**Publisher's note:** Springer Nature remains neutral with regard to jurisdictional claims in published maps and institutional affiliations.

## Supplementary Material

Supplementary Methods and Figures
