# Supplementary material for: Fibroblast growth factor 21, assisted by elevated glucose, activates paraventricular nucleus NUCB2/Nesfatin-1 neurons to produce satiety under fed states
Source: Sci Rep. 2017 Apr 4;7:45819. doi: 10.1038/srep45819 (PMC5379189; doi:10.1038/srep45819)
Supplement: Supplementary Methods and Figures [file srep45819-s1.pdf]

Fibroblast growth factor 21, assisted by elevated glucose, activates paraventricular nucleus NUCB2/Nesfatin-1 neurons to produce satiety under fed states

Putra Santoso, Masanori Nakata, Kazuhiro Shiizaki, Zhang Boyang, Kumari Parmila, Zesemdorj Otgon-Uul, Koshi Hashimoto, Tetsurou Satoh, Masatomo Mori, Makoto Kuro-o and Toshihiko Yada

#Address of correspondence:
Prof. Toshihiko Yada
Department of Physiology, Division of Integrative Physiology, Jichi Medical University, Yakushiji 3311-1, Shimotsuke, Tochigi 329-0498, Japan.
Phone: +81-285-58-7320 /Fax: +81-285-44-9962
E-mail: tyada@jichi.ac.jp

Supplementary method

Establishment of NucB2 floxed mice

Nucb2 floxed mice were generated by homologous recombination using the ES cell line RENKA, which was established from C57BL/6N mouse strain. A targeting vector was constructed as follows (Supplementary Fig. 2A). First, three fragments (the 5' arm, the floxed-out region, and the 3' arm) were subcloned using polymerase chain reaction (PCR) from the BAC clone RP23-383B22 or RP23-329L15. A 5.9-kb region containing exon 3-6 with the DNA-binding paired domain was used for the floxed-out region. Two homologous genomic DNA fragments, 5' and 3' arms, were 3.0 and 5.0 kb in size, respectively. These three PCR products were inserted into a pBS vector containing neomycin resistance (neo) cassette flanked by two Flp recognition target (FRT) sites and two loxP sites. The floxed-out exon 3-6 fragment was inserted between two loxP sites. The diphtheria toxin A fragment (DT-A), driven by the thymidine kinase (tk) promoter, was inserted at the 5' end of the vector. Mouse Bruce-4 ES cells were transfected with the targeting vector by electroporation, and G418-resistant clones were screened for homologous recombination by PCR. Positive clones were injected into blastocysts to generate Nucb2 $^{flox/+}$/NeoFRT mice. NUCB2 $^{flox/+}$/NeoFRT mice were crossed with CAG-FLPe transgenic mice to remove the Neo cassette. Offsprings were backcrossed to wild-type C57BL/6 mice to remove the CAG-FLPe transgene, generating NUCB2 $^{flox/+}$ mice. NUCB2 flox mutant allele was detected using PCR (Supplementary Fig. 2B) with the following primers: 5'-TTCTCCTGTACTTTCCTGTCAACAC -3' and 5'-AGACCCTACCTCAAAACAGTCAGAG -3'. Amplification of wild-type and mutant alleles generates 212- and 391-bp fragments, respectively.

Supplementary Table 1, related to RT-PCR procedures. Forward and reverse primers used in RT-PCR experiments.

Gene	Primer	
	Forward	Reverse
NUCB2	5’-GTCACAAAGTGAGGACGAGACTG-3’	5’-TGGTTCAGGTGTTCAAACTGCTTC-3’
Oxytocin	5’-TGTGCTGGACCTGGATATGCGCA-3’	5’- GGCAGGTAGTTCTCCTCCTG-3’
AVP	5’-CATCTCTGACATGGAGCTGAGA-3’	5’- GGCAGGTAGTTCTCCTCCTG-3’
CRH	5’-TCTCTCTGGATCTCACCTTCCACC-3’	5’-AGCTTGCTGAGCTAACTGCTCTGC-3’
GADPH	5’-GGCACAGTCAAGGCTGAGAATG-3’	5’-ATGGTGGTGAAGACGCCAGTA-3’.

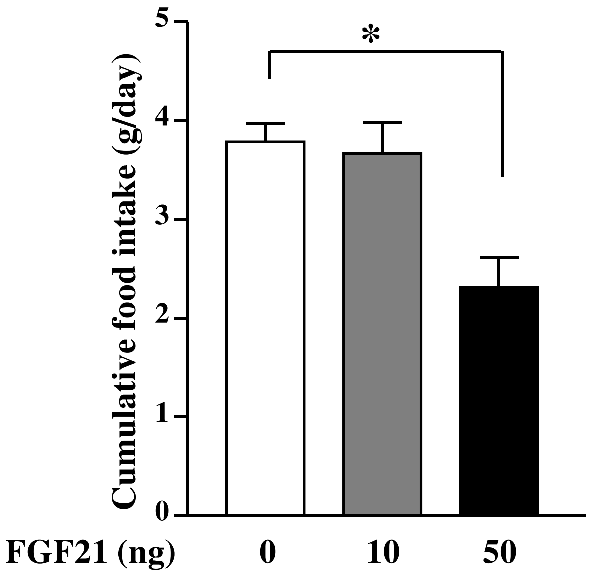

Supplementary Figure 1.
FGF21 dose-dependently reduced food intake. FGF21 at a dose of 10 ng did not alter food intake at 24 h (grey bar), while at 50 ng it significantly reduced food intake at 24 h (black bar). Data are presented as mean ± SEM. *$P < 0.05$ by one-way ANOVA followed by Bonferroni post-hoc test. n = 3-5.

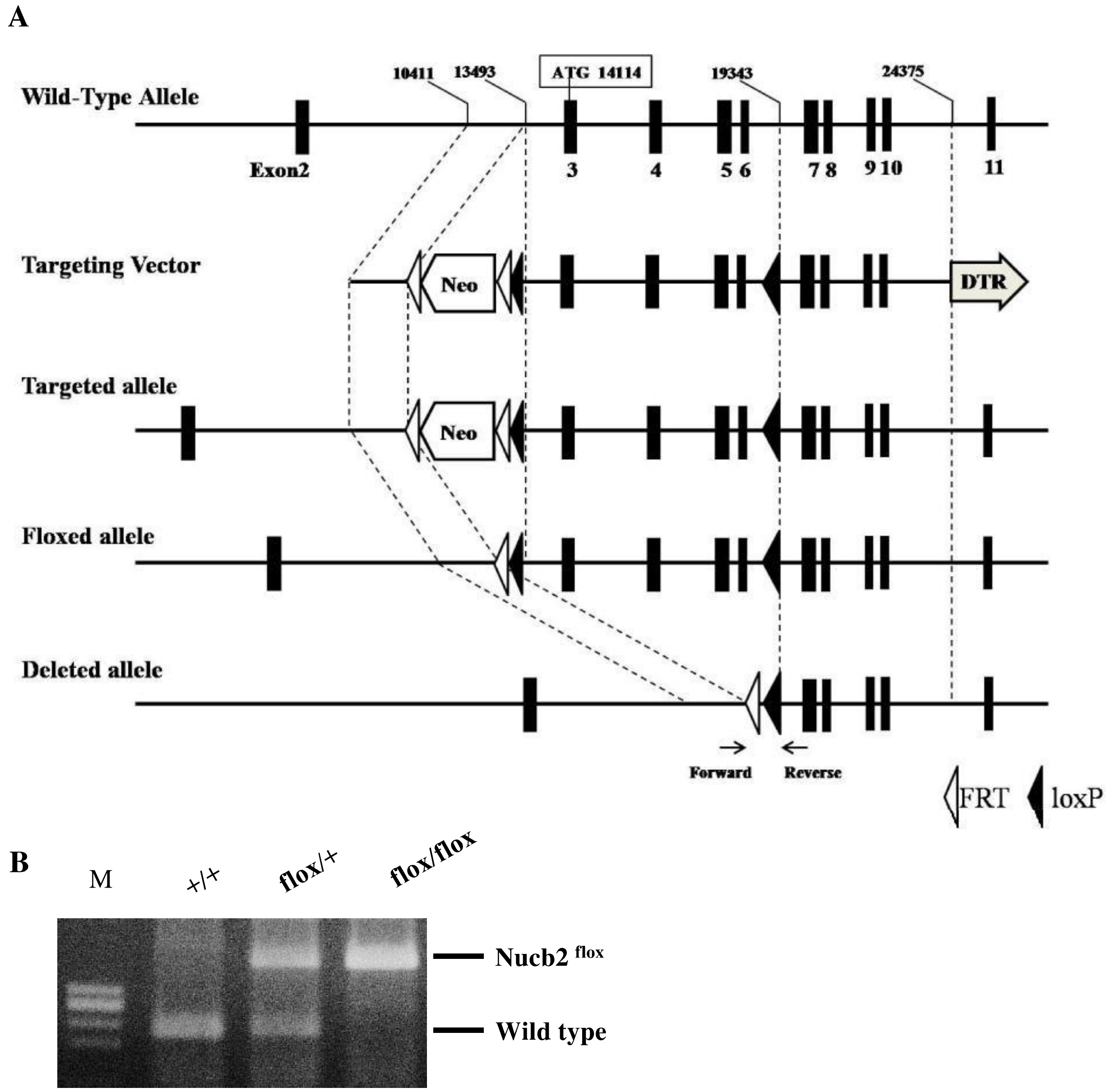

Supplementary Figure 2.

A, Schematic representation of wild-type and mutant NUCB2 alleles together with the targeting vector. Black boxes show exons. In the target vector, a 5.9-kb region containing exon 3-6 and its flanking region were replaced by a 7.6-kb fragment carrying two loxP sequences (filled arrowheads) and PGK-neo (NeoR) flanked by FRT sequences (open arrowheads). Diphtheria toxin A fragment driven by the tk promoter (DTR) was inserted at the 5'site of the vector. In NeoFRT allele, the NUCB2 chromosomal gene was replaced by the targeting vector. In Floxed allele, the FRT-flanked NeoR gene was removed by FLPe recombinase. In deleted allele, the loxP-flanked exon 3-6 of Nucb2 gene was deleted by Cre recombinase. *B*, Genotypes of the mice were determined by PCR. A typical gel image is shown. M : size marker.

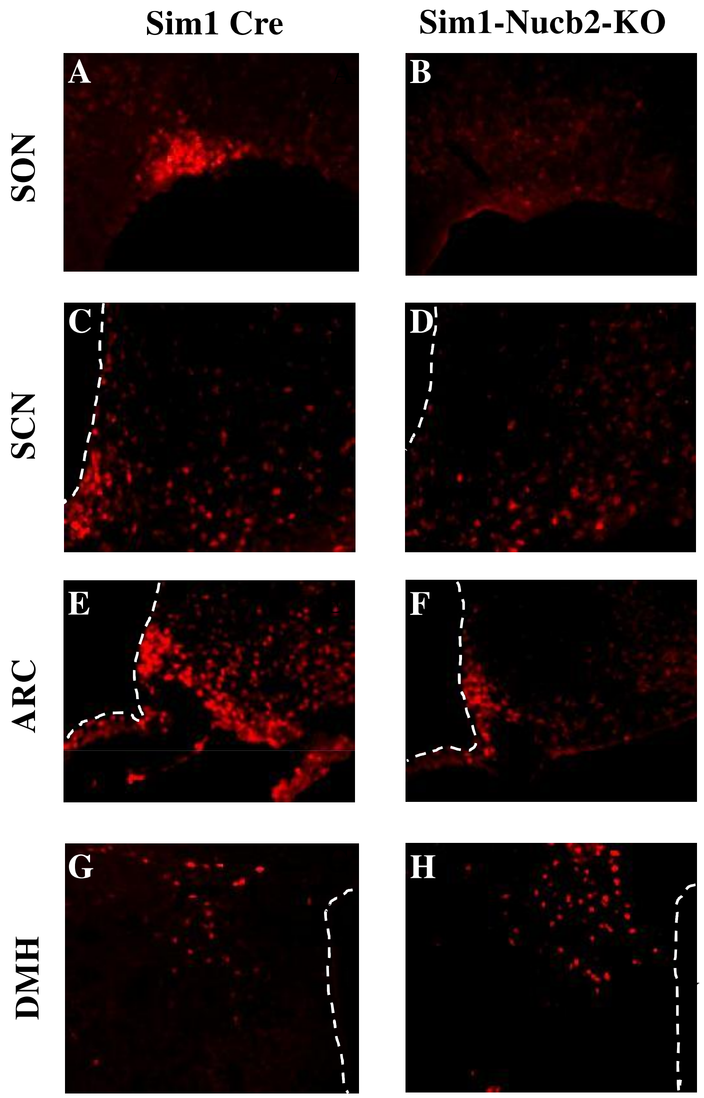

Supplementary Figure 3.

NUCB2/Nesf-1 expression was absent in the SON but remained intact in the SCN, ARC, and DMH of the Sim1-Nucb2-KO mice. Immunofluorescence staining of NUCB2/Nesf-1 in the SON (A, B), SCN (C, D), ARC (E, F), and DMH (G, H) for Sim1 Cre mice and Sim1-Nucb2-KO mice.
